# High value correlates of caregiver reported counseling service need and utilization for adolescents at-risk for childhood maltreatment and neglect

**DOI:** 10.1371/journal.pone.0258082

**Published:** 2021-10-01

**Authors:** Alejandro L. Vázquez, Tommy Chou, Cynthia M. Navarro Flores, Tyson S. Barrett, Miguel T. Villodas, Melanie M. Domenech Rodríguez

**Affiliations:** 1 Department of Psychology, Utah State University, Logan, Utah, United States of America; 2 Department of Psychology, Florida International University, Miami, Florida, United States of America; 3 Department of Psychology, San Diego State University, San Diego, California, United States of America; Temple University, UNITED STATES

## Abstract

Adolescents with a history of child maltreatment experience increased risk for psychopathology that sets them on a trajectory towards a range of difficulties in adulthood. Various factors influence caregivers’ decisions to seek mental health services (MHS) that could improve developmental outcomes. The present study applied a machine learning algorithm, elastic net, to a sample of 878 adolescent-caregiver dyads from the Longitudinal Studies of Child Abuse and Neglect. Analyses simultaneously examined a large number of factors to determine their ability to discriminate between caregivers who perceived a need for MHS and those who did not, as well as caregivers who utilized MHS and those who did not. Results highlight family demographics, chronic parental stressors, youth psychopathology, and exposure to recent adversities as good classifiers of caregiver perceived need for (77.6%; sensitivity = .77; specificity = .78) and utilization of (71%; sensitivity = .71; specificity = .71) adolescent MHS. Elastic net identified adolescent clinical externalizing and internalizing problems, and parental stress related to child(ren)’s behavior as high value classifiers of both outcomes. Youth living with non-kin caregivers were also significantly more likely to utilize MHS. Findings highlight the importance of assessing clinical need, stress related to child(ren)’s behavior, and caregiver kinship in understanding the likelihood that at-risk families will seek adolescent MHS.

## Introduction

A robust body of literature relates child maltreatment with a host of negative correlates including atypical brain development, higher rates of social problems and relationship disruptions, language delays, and poorer quality of life [1]. Perhaps most notably, extensive research highlights the impact of child abuse and neglect on risk for psychopathology. Existing work indicates an increased incidence of internalizing (e.g., anxiety disorders, major depressive disorder, post-traumatic stress disorder) and externalizing (e.g., oppositional defiant disorder, attention-deficit hyperactivity disorder, conduct disorder) disorders among children who have experienced maltreatment [2]. Childhood maltreatment in the U.S. has a low-end estimated economic burden of $428 billion in costs for physical, mental health, and correctional services [3]. Although effective evidence-based interventions exist, many barriers prevent caregivers from utilizing needed mental health services (MHS) for their child during periods in which psychopathology is often present or has begun to emerge, such as adolescence [4]. The current study sought to deepen our understanding of factors associated with caregiver perceived need for and utilization of adolescent MHS through the examination of family demographics, chronic parental stressors, youth psychopathology, and exposure to recent adversities.

### Mental health services utilization

Rigorous empirical research has resulted in the development of various psychosocial prevention and intervention protocols to promote recovery and healthy growth among maltreated children and young adults [5, 6]; however, barriers to care often preclude access to necessary MHS [7]. Broadly, systemic socioeconomic and racial disparities impact service use [8]. Additionally, logistic barriers affect access to care, such as geographic availability of providers, lengthy waitlists, and cost of care further impede families’ treatment-seeking attempts [7]. Further, evidence relates perceived need for services, type of maltreatment, child age and gender, race/ethnicity, foster versus non-foster family status, insurance and Medicaid coverage, and caregiver education to MHS utilization [7, 9, 10]. Caregivers are the primary gatekeepers in mental healthcare among youth and their prior experiences with child welfare services (CWS) are important determinants in their decision-making and treatment-seeking behaviors (e.g., perceptions of unfair treatment and harassment, concerns about child removal [11]). These factors form a complex network that contributes to caregivers’ decisions regarding whether to utilize youth MHS.

Theoretical frameworks of help seeking behavior suggest that families’ decisions to engage in health services begin with problem recognition (i.e., clinical need, caregiver perceived need), which informs subsequent decisions to seek appropriate services [12, 13]. Families must weigh a variety of factors (e.g., finances, transportation, social support, caregiver functioning, insurance) and multiple priorities to determine the feasibility of and need for formal intervention [12]. These conceptual models suggest that research looking to understand caregivers’ help seeking behavior must consider various factors that may account for utilization of MHS. However, traditional statistical methodologies (e.g., multiple linear regression) may be ill-equipped to simultaneously examine large numbers of variables that may provide important information needed to accurately identify families at-risk of underutilization of MHS. Constraints such as multicollinearity and family-wise error rate limit the examination of many correlated predictors [14]. These limitations may pose a challenge to CWS agencies that must consider a wide range of predictors when estimating the likelihood that families will seek recommended MHS for their child [15]. CWS agencies operate in the context of numerous constraints, including increasing caseloads despite stagnant or decreasing funding; thus, many have implemented risk assessments that use predictive models and actuarial tables, which are based on large datasets and advanced analytic techniques, to support frontline providers in more efficiently assessing risk for single incident or recurring maltreatment [16]. Similar efforts utilizing innovative machine learning algorithms and available rich datasets from multisite research may further clarify top priority considerations in needs assessment, referral to services, and follow-up care.

### Machine learning: Elastic net

Recent advancements in computational power have contributed to the emergence of machine learning algorithms that can draw information from broad sets of variables to maximize classification of a particular outcome [17]. Elastic net is an algorithm that combines the strengths of lasso (i.e., variable selection) and ridge (i.e., handling multicollinearity) regressions to select variables for inclusion in sparse models that improve classification of outcomes [18]. Elastic net excels in situations that would hinder traditional statistical methods (e.g., multicollinearity), as it strives for model parsimony while retaining intercorrelated factors that provide unique information and improve classification accuracy [14]. Using this modeling approach, researchers can obtain measures of relative variable importance based on random permutation tests. Variable importance provides information on factors that reduce standard error in classifying dichotomous outcome (e.g., utilized counseling or did not). Information drawn from these analyses may improve providers’ abilities to match families with available MHS through the identification of high value classifiers associated with adolescent MHS need and utilization.

### Present study

Building on previous research identifying factors related to MHS utilization, the present study applied machine learning analyses to data from a multi-site longitudinal study of youth at-risk for victimization by family violence and neglect during early childhood. We used elastic net to (a) examine the relative importance of family demographics, chronic parental stressors, youth psychopathology, and recent adversities in their association with caregiver perceived need for and utilization of adolescent counseling services, and (b) determine the accuracy of these factors in classifying service need and utilization. Each of the variables included in the models have been identified as being theoretically and/or empirically important factors in previous literature. This work extends prior research by clarifying their importance *relative to each other*. Information drawn from this approach may increase our understanding of MHS utilization and improve engagement and retention of at-risk families.

## Materials and methods

### Procedure and sample characteristics

The current study utilized data collected from 1,354 adolescent-caregiver dyads who participated in the Longitudinal Studies of Child Abuse and Neglect (LONGSCAN) [19]. Participants were recruited from five sites in the Southwestern, Northwestern, Eastern, Southern, and Midwestern United States. Sites recruited families who were at-risk for child abuse and neglect (i.e., Eastern), had contact with Child Protective Services (CPS; i.e., Southwestern, Northwestern), or both (i.e., Southern, Midwest). All sites used uniform methods of data collection and entry. Youth-caregiver dyads were interviewed face-to-face and completed self-report measures biannually starting at age 4. Study staff conducted interviews and administered self-report measures using laptop computers. Study representatives at each site reviewed CPS records to identify reports of alleged abuse (i.e., physical, emotional, sexual), domestic violence, and household substance abuse. Coders utilized a modified version of the Maltreatment Classification System (MMCS) and were trained until they reached 90% agreement [20, 21]. Runyan and colleagues provide additional information regarding the LONGSCAN sample and study procedures [22].

Data for the present study included 878 adolescent-caregiver dyads that participated in the age 16 wave of LONGSCAN. Caregivers were approximately 44 years old (*M* = 44.4; *SD* = 10.4), mostly women (91.2%, *n* = 801), racially and ethnically diverse (Black 54.4% [*n* = 478]; White 31.7% [*n* = 278]; Latinx 8.0% [*n* = 70]; other 5.4% [*n* = 47]), and were predominantly biological parents or relatives of the adolescent (i.e., kin; 81.7%, *n* = 717). See Table 1 for additional information regarding caregiver characteristics by outcomes. Adolescents were nearly 16 years old on average (*M* = 15.8; *SD* = 0.5), were roughly evenly split on gender (girls 51.6% [*n* = 453]; boys 48.4% [*n* = 425]) and varied in race/ethnicity (Black 54.4% [*n* = 478]; White 24.4% [*n* = 214]; Latinx 7.2% [*n* = 63]; other 14% [*n* = 123]). In the present sample, 18.1% (*n* = 159) of adolescents were in the clinical range for externalizing and 11.7% (*n* = 103) for internalizing problems based on the Child Behavior Checklist (see description below). See Table 2 for information on adolescent demographics, psychopathology, and recent adversities by outcome.

**Table 1:** Caregiver demographics at age 16 interview by counseling outcomes.

	Total	Perceived need ^a^	*p* value	Utilized ^a^	*p* value
Sample size	878 (100%)	217 (24.7%)		171 (19.5%)	
Sex					
Female	801 (91.2%)	199 (24.8%)		155 (19.4%)	
Male	70 (8.0%)	16 (22.9%)		14 (20.0%)	
Race/ethnicity			< .001		< .001
White	278 (31.7%)	95 (34.2%)		76 (27.3%)	
Black	478 (54.4%)	88 (18.4%)		69 (14.4%)	
Latinx	70 (8.0%)	18 (25.7%)		14 (20.0%)	
Other	47 (5.4%)	14 (29.8%)		11 (23.4%)	
Marital status			.026		
Married	336 (38.3%)	80 (23.8%)		64 (19.0%)	
Single	283 (32.2%)	57 (20.1%)		47 (16.6%)	
Other ^b^	256 (29.2%)	79 (30.9%)		59 (23.0%)	
Secondary			.023		.019
High school or equivalent	669 (76.2%)	178 (26.6%)		142 (21.2%)	
Less than high school	203 (23.1%)	37 (18.2%)		27 (13.3%)	
Post-secondary			.007		.002
None	408 (46.5%)	83 (20.3%)		60 (14.7%)	
Vocational	313 (35.6%)	81 (25.9%)		67 (21.4%)	
Associate or greater	153 (17.4%)	52 (34.0%)		43 (28.1%)	
Household income					
Low	354 (40.3%)	79 (22.3%)		62 (17.5%)	
Middle	243 (27.7%)	75 (30.9%)		58 (23.9%)	
High	260 (29.6%)	61 (23.5%)		49 (18.8%)	
Kin^c^			< .001		< .001
Yes	717 (81.7%)	151 (21.1%)		111 (15.5%)	
No	153 (17.4%)	64 (41.8%)		58 (37.9%)	

Variable frequency is displayed by column for the total and row for outcomes. *p*-values represent statistically significant chi-square test.

^a^ responded yes to outcome.

^b^ separated, divorced, or widowed.

^c^ biological relative of the adolescent.

**Table 2:** Adolescent demographics, psychopathology, and recent adversities at age 16 interview by counseling outcomes.

	Total	Perceived need ^a^	*p* value	Utilized ^a^	*p* value
Sample size	878	217 (24.7%)		171 (19.5%)	
Sex					
Female	453 (51.6%)	108 (23.8%)		84 (18.5%)	
Male	425 (48.4%)	109 (25.6%)		87 (20.5%)	
Race/ethnicity			.003		.017
White	214 (24.4%)	70 (32.7%)		53 (24.8%)	
Black	478 (54.4%)	94 (19.7%)		73 (15.3%)	
Latinx	63 (7.2%)	19 (30.2%)		15 (23.8%)	
Other	123 (14%)	34 (27.6%)		30 (24.4%)	
Externalizing ^b^			< .001		<. 001
Yes	159 (18.1%)	93 (58.5%)		71 (44.7%)	
No	692 (78.8%)	120 (17.3%)		96 (13.9%)	
Internalizing ^b^			< .001		< .001
Yes	103 (11.7%)	66 (64.1%)		54 (52.4%)	
No	748 (85.2%)	147 (19.7%)		113 (15.1%)	
Recent adversities					
Physical abuse			< .001		< .001
Yes	118 (13.4%)	52 (44.1%)		40 (33.9%)	
No	760 (86.6%)	165 (21.7%)		131 (17.2%)	
Sexual abuse			< .001		< .001
Yes	149 (17%)	59 (39.6%)		49 (32.9%)	
No	729 (83%)	158 (21.7%)		122 (16.7%)	
Emotional abuse			.015		.032
Yes	238 (27.1%)	73 (30.7%)		58 (24.4%)	
No	640 (72.9%)	144 (22.5%)		113 (17.7%)	
Neglect			< .001		< .001
Yes	149 (17%)	59 (39.6%)		49 (32.9%)	
No	729 (83%)	158 (21.7%)		122 (16.7%)	
Household violence			< .001		.005
Yes	534 (60.8%)	156 (29.2%)		121 (22.7%)	
No	344 (39.2%)	61 (17.7%)		50 (14.5%)	
Household arrest/jail			< .001		.001
Yes	171 (19.5%)	61 (35.7%)		49 (28.7%)	
No	707 (80.5%)	156 (22.1%)		122 (17.3%)	
Household substance use			< .001		.001
Yes	171 (19.5%)	61 (35.7%)		49 (28.7%)	
No	707 (80.5%)	156 (22.1%)		122 (17.3%)	
Caregiver depression			< .001		
Yes	254 (28.9%)	84 (33.1%)		55 (21.7%)	
No	553 (63%)	109 (19.7%)		96 (17.4%)	

Variable frequency is displayed by column for the total and row for outcomes. *p*-values represent statistically significant chi-square test.

^a^ responded yes to outcome.

^b^ CBCL clinical threshold (i.e., T-score above 63).

LONGSCAN sites obtained approval to survey families from their local institutional review boards. Caregivers/guardians provided written informed consent before each assessment and adolescents provided written assent to participate. Approval to conduct secondary analysis on the publicly available dataset was obtained from the Utah State University institutional review board.

### Measures

#### Demographics

Demographic information regarding child sex and race/ethnicity were collected at age 4. Caregivers reported their race/ethnicity, marital status, highest completed level of education, relation to the adolescent, employment status, and household income during the age 16 interview. Several demographic variables were condensed to reduce sparsity. Caregiver and adolescent race/ethnicity were recoded into four levels: *White*, *Black*, *Latinx*, *other* (i.e., Native American, Asian or Pacific Islander, Mixed, other). Marital status was recorded into three levels: *married*, *single*, or *separated/divorced/widowed*. Two variables were created to represent whether a caregiver attained a high school diploma or equivalent (i.e., *yes* or *no*) and post-secondary training (i.e., *none*, *vocational*, *associate or greater*). A dichotomous variable was created to represent whether the caregiver was a biological parent or relative of the adolescent (i.e., *kin*, including biological parents, aunts, uncles, grandparents, etc.; versus *non-kin* caregivers such as non-biological foster parents). An indicator was created to represent whether families reported incomes that fell below the federal poverty limit based on their number of dependents during the year the age 16 interview was conducted. The remaining 57% were divided into *medium* and *high* income groups using a median split.

#### Perceived need for and utilization of adolescent counseling

Caregivers’ responded *yes* or *no* to questions examining perceived need for adolescent MHS (i.e., “In the last year, did [child’s name] ever need any type of counseling or therapy, outside of school, for a psychological or behavioral problem?”), and if these services were utilized (i.e., “Did [child’s name] get this professional service, in the last year?”).

#### Externalizing and internalizing behavior

The Child Behavior Checklist is a 113-item questionnaire that assesses a broad variety of adolescent behavioral problems (CBCL) [23]. Parents reported the frequency of their adolescent’s problem behavior in the last six months on a 3-point scale: *not true*, *sometimes true*, or *often true*. CBCL items form two composite scores representing adolescent internalizing (e.g., depression, anxiety) and externalizing problems (e.g., aggression, delinquency). According to standardized scoring procedures, dichotomous variables were calculated to indicate whether adolescents have or do not have clinically elevated internalizing and externalizing problems (i.e., T-score above 63). The CBCL had excellent internal consistency within the current sample for internalizing (α = .90) and externalizing (α = .94) problems scales.

#### Caregiver stressors

The Everyday Stressors Index (ESI) is a 20-item questionnaire designed to assess chronic daily stress among caregivers. LONGSCAN adapted items from a prior measure of caregiver stress (Daily Hassles Scale) to assess various types of chronic stressors: role overload, financial concerns, parenting worries, employment problems, and interpersonal conflict [24]. Caregivers’ responses were: *not at all bothered*, *a little bothered*, *somewhat bothered*, or *bothered a great deal*. While the ESI had good overall internal consistency within the current sample (α = .86), we examined responses at the item level to gain a nuanced understanding of specific caregiver stressors associated with adolescent counseling outcomes that could be obscured through the use of a global score.

#### Recent adversities

Eight dichotomous indicators were used to represent adolescent exposure to recent adversities, similar to those included in recent studies [25, 26], prior to the age 16 interview, including: household illicit substance use, family member incarceration, witnessing violence involving a family member, caregiver depression, neglect, physical abuse, sexual abuse, and emotional abuse. Indicators were derived using all available data from youth and caregiver-report items, as well as CPS reports. We used indicators of CPS allegations of maltreatment based on findings that suggest that children with alleged and substantiated maltreatment have similar risk for developing mental health and behavioral problems [27, 28].

*Physical abuse*. A dichotomous indicator for adolescent physical abuse was derived from caregiver and adolescent self-report, and CPS reports of physical abuse assessed at age 16 (i.e., *no* or *yes*). Endorsement of any of the following scales was coded as an adolescent who experienced physical abuse prior to the age 16 interview. Caregivers completed the Conflict Tactics Scales-Parent-Child version, which provided information on instances of severe (three items; e.g., hit child with fist or kicked hard) and extreme (four items; e.g., burned or scalded child on purpose) assault within the last year [29]. Adolescents reported on 12 dichotomous items (i.e., *no* or *yes*) assessing a variety of caregiver physical abuse behaviors since age 13 (e.g., cut or stabbed, shot at with a gun, punch with their hand), which were based on physical abuse definitions used in the MMCS [20, 21]. Youth with an endorsement from caregivers or themselves, or a CPS allegation between ages 14–16 interviews were coded as having experienced physical abuse.

*Sexual abuse*. A dichotomous indicator for adolescent sexual abuse was derived from adolescent self-reports and CPS reports of sexual abuse assessed at age 16 (i.e., *no* or *yes*). Adolescents reported sexually abusive caregiver behaviors since age 13 on 11 dichotomous items (i.e., *no* or *yes*; e.g., looking at private parts in sexual way, kiss or put mouth on parts or breast on purpose), which drew from sexual abuse definitions used in the MMCS [20, 21]. Youth who endorsed any of these items or had a CPS allegation between ages 14–16 interviews were coded as having experienced sexual abuse.

*Emotional abuse*. A dichotomous emotional abuse indicator was derived from adolescent and CPS reports of emotional abuse assessed as part of the age 16 interview (i.e., *no* or *yes*). Adolescents reported emotionally abusive caregiver behaviors since age 13 on 12 dichotomous items (i.e., *no* or *yes*; e.g., threatened or destroyed things important to the adolescent, scared or upset adolescent by placing them in a situation where they may be hurt), which drew from emotional abuse definitions used in the MMCS [20, 21]. Youth who endorsed any of these items or had a CPS allegation between ages 14–16 interviews were coded as having experienced emotional abuse.

*Neglect*. A dichotomous indicator of adolescent neglect was derived from CPS reported allegations between age 14–16 interviews as there were no self-reported measures that provided clear distinctions between neglected and non-neglected youth (i.e., *no* or *yes*).

*Witnessed violence involving a family member*. A dichotomous indicator representing whether or not adolescents witnessed violence that involved a family member was derived from caregiver self-report and whether domestic violence was indicated as a risk factor in CPS allegations that adolescents received prior to the age 16 interview. Caregivers reported on the Adult Violence in the Home questionnaire, which consisted of six dichotomous items (i.e., *no* or *ye*s) describing increasingly serious acts of violence between adults in the home ranging from serious threats of violence to someone getting shot or raped within the last year [19]. Caregivers who endorsed any of these items or had a CPS allegation that included domestic violence between ages 14–16 interviews were coded as having witnessed family violence.

*Family member arrested or jailed*. A dichotomous indicator was created to represent whether a member of an adolescent’s household had been arrested or in jail within the last year as reported at the age 16 interview (i.e., *no* or *yes*). This indicator was derived from caregiver report on the Child’s Life Events questionnaire, which includes two dichotomous items assessing whether members of the adolescent household had been arrested (i.e., “Was anyone in the household arrested?”) or jailed (i.e., “Was anyone in the household jailed?”) during the last year (i.e., *no* or *yes*) [30]. Endorsement of either of these items were coded as exposure to a household member arrest and/or jailing prior to the age 16 interview.

*Household substance use*. A dichotomous indicator of exposure to household illicit substance use was derived from adolescent self-report and whether drug or alcohol use was indicated as a risk factor in any of the CPS allegations that adolescents received prior the age 16 interview (i.e., *no* or *yes*). Adolescents completed the Risk Behaviors of Family and Friends questionnaire, which included six dichotomous items (i.e., *no* or *yes*) assessing whether they had witnessed a member of their household use illicit drugs (i.e., marijuana, cocaine/crack, meth/speed, inject drugs, use other drugs) and/or get drunk or high [31]. Youth who endorsed any of these items or had a CPS allegation that included alcohol or drug use as a risk factor between ages 14–16 interviews were coded as having household substance use.

*Caregiver depression*. During the age 16 interview, caregivers completed the Center for Epidemiologic Studies Depression Scale (CES-D), which consists of 20 items assessing symptoms associated with depression (e.g., depressed mood, guilt, worthlessness, hopelessness, issues with sleep) within the past week [32]. Internal consistency for the CES-D was excellent (α = .90) within the current sample. The recommended clinical cut-off of 16 was used to distinguish between caregivers who had clinical level depression and those who did not (i.e., *no* or *yes*) [31].

### Analytic plan

Within the current sample, 18.6% (*n* = 163) of participants were missing at least one covariate. Missingness was addressed through mode imputation, which replaced missing values for each variable with the mode. Mode imputation is commonly used within the context of machine learning as analyses focus on classification accuracy based on random permutation tests rather than coefficients and *p* values that can be biased by this method [17, 33].

Chi-squared tests of independence were used to determine whether caregiver perceived need for and utilization of counseling services for their adolescents differed by demographic characteristics, specific youth psychopathology, and recent adversities. The elastic net algorithm was then trained on 39 variables (i.e., demographics, parental stressors, youth psychopathology, recent adversities) to classify counseling service need and utilization within the last year [17]. Algorithm performance was assessed on individual cases using leave-one-out cross-validation (LOOCV) to calculate classification accuracy and prevent overfitting [33]. LOOCV removes a case at random from the data, trains elastic net on the remaining cases, and tests the algorithm’s classification accuracy on the excluded case. LOOCV repeats this process until each case in the sample is excluded and used to validate the algorithm. Classification probability thresholds were then tuned so that accuracy metric would reflect a balance between sensitivity (i.e., probability of true positive) and specificity (i.e., probability of true negative) [17]. Variables were ranked based on their relative importance, which represents the sum of the decrease in classification error when a variable is included in the model as determined by cross-validation. High value classifiers are identified through visual inspection of relative importance figures. Variables that demonstrate large increase in relative importance over subsequent covariates are known as high value classifiers [33]. High value classifiers were examined using a crosstabulation visualization to determine the nature of the relationship between each high-value classifier and the corresponding outcome [34].

## Results

### Descriptive analyses

Perceived need for adolescent counseling services was greater among caregivers who were White (34.2%; *n* = 95), separated/divorced/widowed (30.9%; *n* = 95), had a high school diploma or equivalent (26.6%; *n* = 178), Associate’s degree or greater (34%; *n* = 52), and were non-kin (41.8%; *n* = 64). Utilization of adolescent counseling services was greater among caregivers who were White (27.3%; *n* = 76), had a high school diploma or equivalent (21.2%; *n* = 142), earned an Associate’s degree or greater (28.1%; *n* = 43), and were non-kin (37.9%; *n* = 58). Counseling utilization did not significantly differ by caregiver sex or income. See Table 1 for caregiver demographics grouped by outcomes.

Caregiver-reported perceived need for and utilization of counseling services did not significantly differ by adolescent sex. Caregivers of White adolescents reported greater need (*n* = 70; 32%) and utilization (*n* = 53; 24.8%) of MHS. Caregivers who reported clinical levels of youth externalizing and internalizing problems reported higher rates of perceived need for and utilization of adolescent counseling services. Caregivers who perceived a need for adolescent counseling reported higher rates of service utilization (77%; χ^2^ [1, *n* = 878] = 576.51, *p* < 0.001). Adolescents within the current sample experienced 2.33 (*SD* = 1.82) adversities on average leading into the age 16 interview. Recent adversities were generally associated with greater caregiver-reported need and utilization of counseling services, with the exception of caregiver depression which was not significantly associated with the utilization of counseling services. See Table 2 for adolescent demographics, psychopathology, and recent adversities by counseling outcomes.

### Perceived need

**Fig 1:**
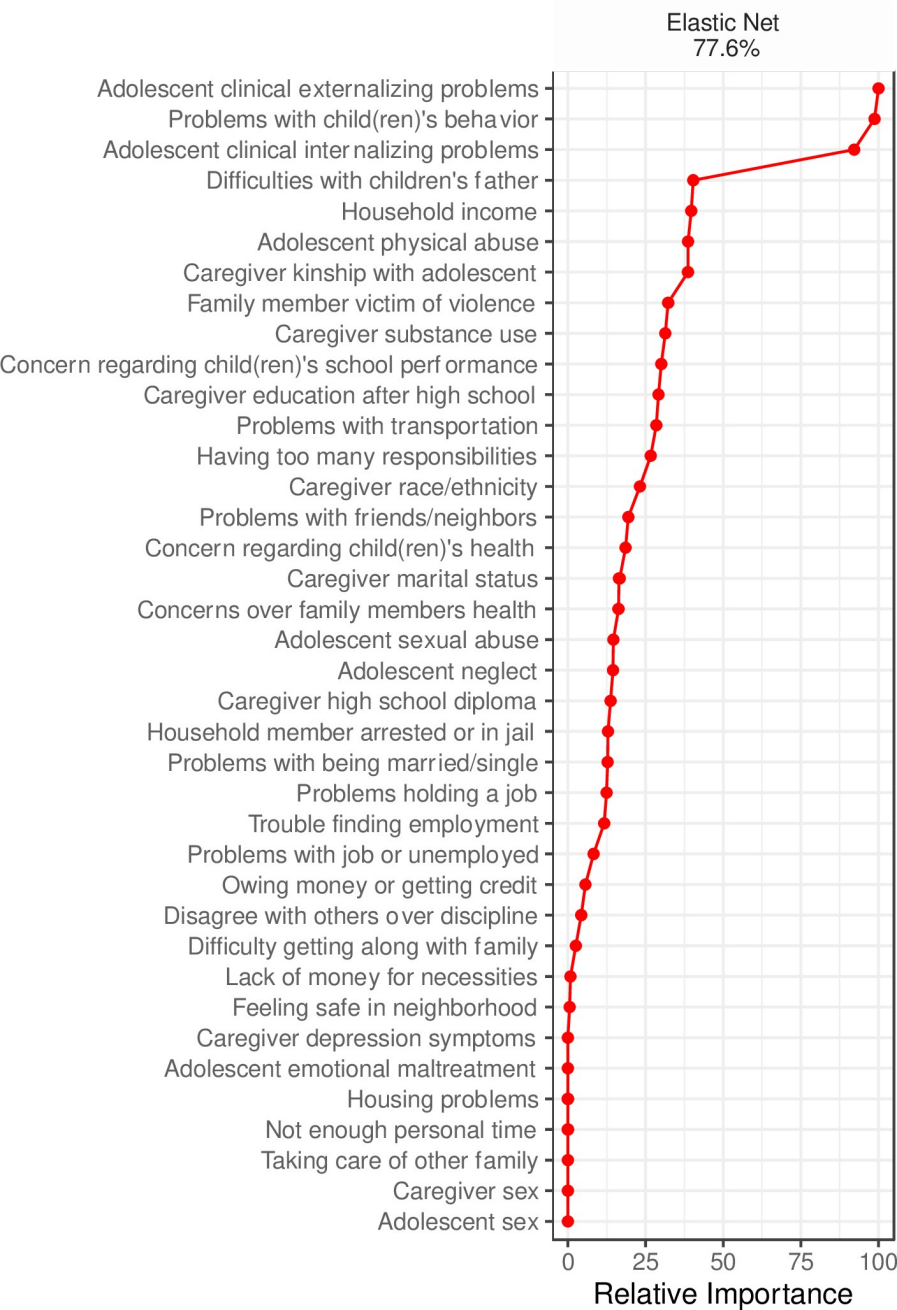
Relative importance of predictors of caregiver perceived need for adolescent counseling services. Predictors that were reduced to zero are not ranked in order of importance. % represents balanced classification accuracy.

Within the current sample, 24.7% of caregivers (*n* = 217) reported perceiving a need for adolescent counseling services in the last year. Elastic net identified a variety of factors that assisted the algorithm in discriminating between caregivers who perceived a need for adolescent counseling services and those who did not. See Fig 1 for variable importance for perceived need for adolescent counseling. Visual inspection of the relative importance plot suggested that clinically-elevated externalizing problems, caregiver stress regarding their child(ren)’s behavior problems, and adolescent clinically-elevated internalizing problems were by far the top classifiers of service need. These factors classified service need well (accuracy = 77.6%; sensitivity = .77; specificity = .78), providing confidence in the importance of these high value factors. Crosstabulation visualizations suggest that caregiver stress related to child(ren)’s behavior (i.e., *a little bothered*, *somewhat bothered*, *bothered a great deal*) and adolescent clinically-elevated internalizing/externalizing problems were associated with greater reported perceived need for services. S1 Fig shows important variable mosaics for service need.

### Utilization

**Fig 2:**
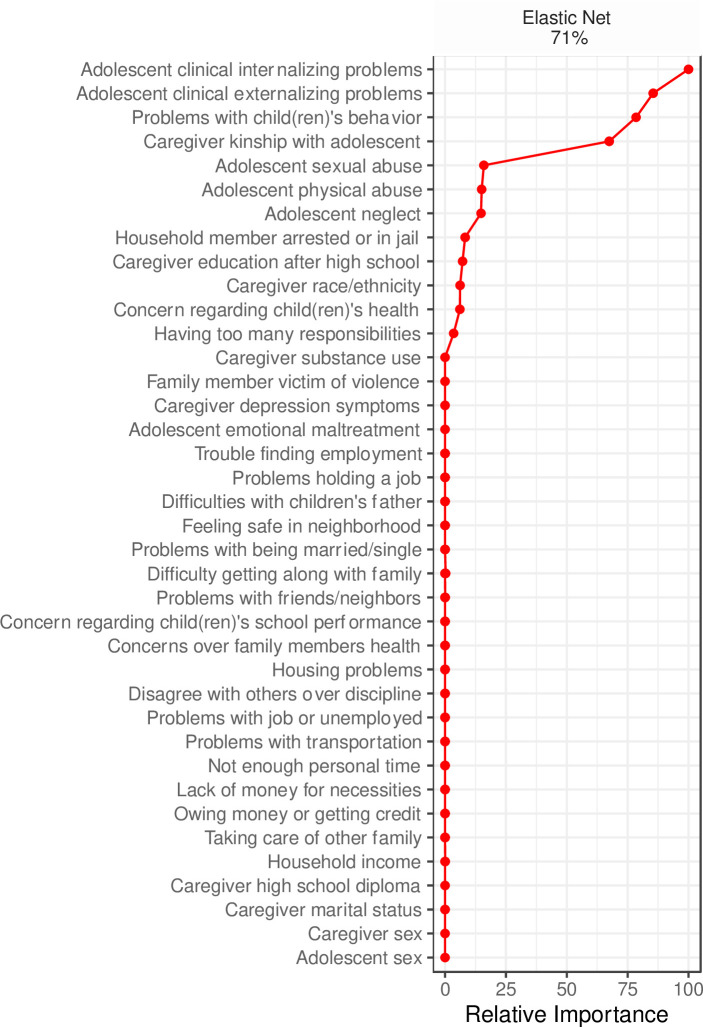
Relative importance of predictors of caregiver utilization of adolescent counseling services. Predictors that were reduced to zero are not ranked in order of importance. % represents balanced classification accuracy.

Within the current sample, 19.5% (*n* = 171) of caregivers reported utilizing adolescent counseling services in the last year. Elastic net selected fewer variables that aided the classification of service utilization relative to need. See Fig 2 for variable importance in classifying adolescent counseling utilization. Elastic net identified youth clinically elevated internalizing problems as the top classifiers of caregiver utilization of adolescent counseling services. Internalizing problems was followed in importance by clinically elevated externalizing problems, caregiver stress related to problems with youth behavior, and caregiver kinship with the adolescent. Elastic net accurately classified 71% (sensitivity = .71; specificity = .71) of caregiver reported utilization of adolescent counseling services. Crosstabulation visualizations suggest that adolescent clinical externalizing/internalizing problems, being a non-kin caregiver, and stress related to child(ren)’s behavior (i.e., *a little bothered*, *somewhat bothered*, *bothered a great deal*) were associated with a greater proportion of reported service utilization. See S2 Fig for important variable mosaics for service need.

## Discussion

The current study expands the literature through the identification of high value classifiers associated with caregiver reported need for and utilization of psychological counseling services for adolescents at-risk for childhood maltreatment and neglect. Algorithms utilizing information on family demographics, chronic caregiver stressors, youth psychopathology, and exposure to recent adversities achieved high levels of classification accuracy for caregiver perceived need for and utilization of adolescent counseling services. Elastic net identified adolescent symptomatology and parenting stress related to adolescents’ behavior as the most important discriminators between caregivers who perceived a need for and utilized adolescent counseling services and those that did not. Having a non-kin caregiver was specifically important in classifying service utilization. These findings may represent the importance of adolescent psychopathology and related parenting stress in alerting and mobilizing caregivers towards seeking formal MHS for their youth.

While previous research has identified youth psychopathology and parenting stress related to youth’s behavioral challenges as factors associated with child MHS utilization [7], our findings suggest that these factors have greater discriminatory importance relative to traditional indicators of mental health disparities in classifying caregiver perceived need for and utilization of adolescent counseling services (e.g., transportation, finances, race/ethnicity, adversity) [8, 35, 36]. These findings suggest that when compared to other factors, adolescents’ mental health challenges and associated parenting stress may be the primary driving forces determining whether at-risk families access adolescent counseling services. Our findings also confirm the significance of caregiver kinship as an important discriminator between adolescents who accessed counseling services and those that do not [7]. Prior research has documented lower rates of MHS utilization among children living with biological parents or relatives versus those with non-kin caregivers [15] and our findings suggest that this difference may persist into adolescence. Youth living with kin caregivers may be less likely to access MHS as these families receive less monitoring from CWS, have less funding and support for services, and fewer chances for follow ups by caseworkers relative to adolescents in out-of-home-placements [37].

While some factors did not rise to the importance of high value classifiers in our analyses, this does not suggest that they did not provide useful information regarding counseling outcomes. Notably, elastic net had lower classification accuracy and selected fewer variables as providing information regarding counseling utilization relative to those for perceived need. These findings suggest that families may consider a wider range of factors when determining whether their adolescent’s problems *need* formal intervention relative to subsequent stages where they *utilize* MHS. Our findings support the notion that families weigh multiple variables during the problem recognition stage (i.e., perceived need) to determine whether the adolescent problems are sufficiently important and of a magnitude requiring formal intervention [12, 13].

### Implications

Professional evaluation of clinical need and referral to therapy is a common practice of alerting families to the need for formal adolescent MHS [38]. Our findings highlight the importance of assessing clinical need, caregiver stress related to their child(ren)’s behavior, and kindship when engaging at-risk families in adolescent counseling services. Using these high-value indicators, CWS providers may better determine which at-risk families need additional follow-up and support towards identifying and engaging in MHS for adolescents in their care. Furthermore, our findings suggest that caregiver stress associated with adolescents’ challenges is an important correlate of caregivers’ treatment-seeking behaviors; thus, alerting caregivers to the notion that therapy may alleviate youth psychopathology and related caregiver stress may increase their perceived benefits of and motivation to engage in adolescent counseling [39]. Our findings also highlight the need to identify and address potential barriers to accessing adolescent counseling services among at-risk youths living with caregivers who are their biological parent or relative.

Variables examined within the current study were better classifiers of perceived need relative to utilization of adolescent MHS. This difference may ultimately point to factors that help CWS providers more accurately identify caregivers’ stage in the process of help seeking that are appropriate for intervention (i.e., intention versus action as an example aligned with the Theory of Planned Behavior) [40]. Our findings suggest that the problem recognition stage (i.e., perceived need) may be a promising time for CWS providers to implement interventions aiming to promote access to adolescent counseling services [13]. Caregivers’ perceived need for services is mutable and can be increased through psychoeducation [12]. This understanding coupled with clinical skills targeting decision making and behavioral change, such as Motivational Interviewing, may support frontline workforces in more effectively directing families towards care. Engaging kin caregivers in a program like the Family Check-Up (FCU) could provide additional monitoring and function as a gateway to youth MHS on as needed-basis [41]. The FCU intervention provides referrals to youth MHS based on an ecological family assessment and reduces ambivalence towards help seeking through the use of Motivational Interviewing techniques [42].

### Limitations

Findings from the current study should be viewed within the context of several limitations. The algorithm used by the current study do not imply causal mechanisms explaining service need/utilization but rather identified factors that are strong classifiers of group membership (e.g., utilized or did not). The current study utilized cross-sectional data collected as part of the age 16 wave of LONGSCAN. Thus, the causal order between classifiers and outcomes could not be established. Research is needed to examine important classifiers identified by the current study longitudinally to determine whether they predict caregiver reported counseling service need and utilization across time. Importantly, it bears mentioning that while results from the current study speak to high value correlates of caregivers’ perceived need for and utilization of MHS for *at-risk youth*, they do not provide insight as to the specific variables most important in determining whether or not caregivers detect a need for and seek treatment for adolescents *who meet clinical criteria for psychopathology*. Keeping this limitation in mind may consolidate the fact that we did not identify demographic factors such as income or ethnic/racial minority status as high value contributors, with existing literature that speaks to the disparities in MHS access experienced by families in these communities [8, 15].

Researchers may improve upon our classification accuracy through (a) the use of larger datasets with a greater number of positive class examples, (b) utilization of more robust performance metrics/validation (e.g., area under the receiver operator characteristic curve, 10-fold cross-validation, separate validation test set), and (c) the inclusion of additional variables known to impact mental health service utilization (e.g., stigma, shame, discrimination, health insurance). Researchers may also consider utilizing a wider variety of classification algorithms to identify those that best capture mental health service use (e.g., support vector machines, random forest, neural networks gradient boosting machines). Future researchers may also consider utilizing machine learning to examine predictors of other services that caregivers may access to address youth psychopathology (e.g., telepsychology, mentorship programs, parenting classes) [43, 44].

## Conclusions

Existing research provides a broad understanding of factors that impact the utilization of necessary MHS among youth who have experienced maltreatment and neglect; however, the resource scarcity of and high demand on CWS agencies and the larger systems within which they operate make it difficult to glean actionable insights from current science without prioritization for efficiency. Elastic net—and machine learning coupled with large, nuanced datasets in general—shows promise in further advancing the field by highlighting new areas of study and bridging the research-to-practice gap. Our findings suggest that caregivers are likely to seek services for psychopathology, especially when they are experiencing stress related to managing their adolescent’s behavior. The current study also highlights the need to identify and address potential barriers to accessing adolescent counseling services among at-risk youth living with their biological family relative to those in out-of-home care. This information may assist stakeholders in more efficiently engaging adolescents at-risk for maltreatment and neglect in counseling services.

## Supporting information

S1 Fig Crosstab visualization of important classifiers: Perceived need for adolescent counseling services. (TIF)

S2 Fig Crosstab visualization of important classifiers: Utilization of adolescent counseling services. (TIF)

## References

[1] Cicchetti D. Socioemotional, personality, and biological development: Illustrations from a multilevel developmental psychopathology perspective on child maltreatment. Annu Rev Psychol. 2016;67: 187–211. doi: 10.1146/annurev-psych-122414-033259 26726964

[2] Jaffee SR. Child Maltreatment and Risk for Psychopathology in Childhood and Adulthood. Annu Rev Clin Psychol. 2017;13: 525–551. doi: 10.1146/annurev-clinpsy-032816-045005 28375720

[3] Peterson C, Florence C, Klevens J. The economic burden of child maltreatment in the United States, 2015. Child Abus Negl. 2018;86: 178–183. doi: 10.1016/j.chiabu.2018.09.018 30308348 PMC6289633

[4] De Girolamo G, Dagani J, Purcell R, Cocchi A, McGorry PD. Age of onset of mental disorders and use of mental health services: Needs, opportunities and obstacles. Epidemiol Psychiatr Sci. 2012;21: 47–57. doi: 10.1017/s2045796011000746 22670412

[5] Hamilton-Giachritsis C. What Helps Children and Young People Move Forward Following Child Maltreatment? Child Abus Rev. 2016;25: 83–88. doi: 10.1002/car

[6] Wilson CA. Special Issue of Child Maltreatment on Implementation: Some Key Developments in Evidence-Based Models for the Treatment of Child Maltreatment. Child Maltreat. 2012;17: 102–106. doi: 10.1177/1077559512436680 22357754

[7] Staudt MM. Mental Health Services Utilization by Maltreated Children: Research Findings and Recommendations. Child Maltreat. 2003;8: 195–203. doi: 10.1177/1077559503254138 12934636

[8] Maguire-Jack K, Cao Y, Yoon S. Racial disparities in child maltreatment: The role of social service availability. Child Youth Serv Rev. 2018;86: 49–55. doi: 10.1016/j.childyouth.2018.01.014

[9] Kang J. Pathways from social support to service use among caregivers at risk of child maltreatment. Child Youth Serv Rev. 2012;34: 933–939. doi: 10.1016/j.childyouth.2012.01.024

[10] Palusci VJ. Risk factors and services for child maltreatment among infants and young children. Child Youth Serv Rev. 2011;33: 1374–1382. doi: 10.1016/j.childyouth.2011.04.025

[11] Palmer S, Maiter S, Manji S. Effective intervention in child protective services: Learning from parents. Child Youth Serv Rev. 2006;28: 812–824. doi: 10.1016/j.childyouth.2005.08.010

[12] Anderson RM. Revisiting the Behavioral Model and Access to Medical Care: Does it Matter? 1995;36: 1–10. doi: 10.2307/2137284 7738325

[13] Cauce AM, Domenech-Rodríguez M, Paradise M, Cochran BN, Shea JM, Srebnik D, et al. Cultural and contextual influences in mental health help seeking: A focus on ethnic minority youth. J Consult Clin Psychol. 2002;70: 44–55. doi: 10.1037//0022-006x.70.1.44 11860055

[14] Breiman L. Statistical modeling: The two cultures. Stat Sci. 2001;16: 199–215. doi: 10.1214/ss/1009213726

[15] Leslie LK, Hurlburt MS, James S, Landsverk J, Slymen DJ, Zhang J. Relationship between entry into child welfare and mental health service use. Psychiatr Serv. 2005;56: 981–987. doi: 10.1176/appi.ps.56.8.981 16088016 PMC1519415

[16] Cuccaro-Alamin S, Foust R, Vaithianathan R, Putnam-Hornstein E. Risk assessment and decision making in child protective services: Predictive risk modeling in context. Child Youth Serv Rev. 2017;79: 291–298. doi: 10.1016/j.childyouth.2017.06.027

[17] James G, Witten D, Hastie T, Tibshirani R. An Introduction to Statistical Learning. Current Medicinal Chemistry. New York, NY: Springer New York; 2013. doi: 10.1007/978-1-4614-7138-7

[18] Barrett TS, Lockhart G. Efficient Exploration of Many Variables and Interactions Using Regularized Regression. Prev Sci. 2019;20: 575–584. doi: 10.1007/s11121-018-0963-9 30506295

[19] Runyan D, Dubowitz H, English DJ, Kotch JB, Litrownik A, Thompson R, et al. Longitudinal Studies of Child Abuse and Neglect (LONGSCAN) Assessments 0–14. 2011. Available: https://www.ndacan.acf.hhs.gov/datasets/dataset-details.cfm?ID=158

[20] Barnett D, Manly J, Cicchetti D. Defining child maltreatment: The interface between policy and research. In: Cicchetti D, Toth SL, editors. Advances in applied developmental psychology: Child abuse, child development and social policy. Ablex Publishing Corp; 1993. pp. 1–10.

[21] English D, Investigators L. Modified maltreatment classification system (MMCS). 1997.

[22] Runyan DK, Curtis PA, Hunter WM, Black MM, Kotch JB, Bangdiwala S, et al. Longscan: A consortium for longitudinal studies of maltreatment and the life course of children. Aggress Violent Behav. 1998;3: 275–285. doi: 10.1016/S1359-1789(96)00027-4

[23] Achenbach T. Integrative manual for the 1991 CBCL/4-18, YSR, and TRF profiles. Dep Psychiatry, Univ Vermont.

[24] Kanner AD, Coyne JC, Schaefer C, Lazarus RS. Comparison of two modes of stress measurement: Daily hassles and uplifts versus major life events. J Behav Med. 1981;4: 1–39. doi: 10.1007/BF00844845 7288876

[25] Felitti VJ, Anda RF, Nordenberg D, Williamson DF, Spitz AM, Edwards V, et al. REPRINT OF: Relationship of Childhood Abuse and Household Dysfunction to Many of the Leading Causes of Death in Adults: The Adverse Childhood Experiences (ACE) Study. Am J Prev Med. 2019;56: 774–786. doi: 10.1016/j.amepre.2019.04.001 31104722

[26] García BH, Vázquez AL, Moses JO, Cromer KD, Morrow AS, Villodas MT. Risk for substance use among adolescents at-risk for childhood victimization: The moderating role of ADHD. Child Abuse Negl. 2021;114: 104977. doi: 10.1016/j.chiabu.2021.104977 33578244

[27] Hussey JM, Marshall JM, English DJ, Knight ED, Lau AS, Dubowitz H, et al. Defining maltreatment according to substantiation: Distinction without a difference? Child Abus Negl. 2005;29: 479–492. doi: 10.1016/j.chiabu.2003.12.005 15970321

[28] Kohl PL, Jonson-Reid M, Drake B. Time to Leave Substantiation Behind. Child Maltreat. 2009;14: 17–26. doi: 10.1177/1077559508326030 18971346

[29] Straus MA, Hamby SL, Finkelhor D, Moore DW, Runyan D. Identification of Child Maltreatment With the Parent-Child Conflict Tactics Scales: Development and Psychometric Data for a National Sample of American Parents. Child Abuse Negl. 1998;22: 249–270. doi: 10.1016/s0145-2134(97)00174-9 9589178

[30] Coddington RD. The significance of life events as etiologic factors in the diseases of children—II a study of a normal population. J Psychosom Res. 1972;16: 205–213. doi: 10.1016/0022-3999(72)90045-1 5072914

[31] Hunter WM, Cox CE, Teagle S, Johnson RM, Mathew R, Knight ED, et al. Measures for assessment of functioning and outcomes in longitudinal research on child abuse. 2003. Available: http://www.iprc.unc.edu/longscan/

[32] Radloff LS. The CES-D Scale: A Self-Report Depression Scale for Research in the General Population. Appl Psychol Meas. 1977;1: 385–401. doi: 10.1177/014662167700100306 26918431

[33] Hastie T, Tibshirani R, Friedman J. The Elements of Statistical Learning. Second. New York: Springer; 2013. doi: 10.1007/978-1-4419-9863-7_941

[34] Vázquez AL, Domenech Rodríguez MM, Barrett TS, Schwartz S, Amador Buenabad NG, Bustos Gamiño MN, et al. Innovative Identification of Substance Use Predictors: Machine Learning in a National Sample of Mexican Children. Prev Sci. 2020;21: 171–181. doi: 10.1007/s11121-020-01089-4 31960262

[35] Chartier MJ, Walker JR, Naimark B. Separate and cumulative effects of adverse childhood experiences in predicting adult health and health care utilization. Child Abus Negl. 2010;34: 454–464. doi: 10.1016/j.chiabu.2009.09.020 20409586

[36] Fawley-King K, Haine-Schlagel R, Trask E V., Zhang J, Garland AF. Caregiver participation in community-based mental health services for children receiving outpatient care. J Behav Heal Serv Res. 2013;40: 180–190. doi: 10.1007/s11414-012-9311-1 23250770 PMC3625670

[37] Leslie LK, Landsverk J, Ezzet-Lofstrom R, Tschann JM, Slymen DJ, Garland AF. Children in foster care: Factors influencing outpatient mental health service use. Child Abus Negl. 2000;24: 465–476. doi: 10.1016/s0145-2134(00)00116-2 10798837

[38] Alegría M, Lin JY, Green JG, Sampson NA, Gruber MJ, Kessler RC. Role of Referrals in Mental Health Service Disparities for Racial and Ethnic Minority Youth. J Am Acad Child Adolesc Psychiatry. 2012;51: 703–711.e2. doi: 10.1016/j.jaac.2012.05.005 22721593 PMC3652396

[39] Cooley ME, Veldorale-Griffin A, Petren RE, Mullis AK. Parent-Child Interaction Therapy: A Meta-Analysis of Child Behavior Outcomes and Parent Stress. J Fam Soc Work. 2014;17: 191–208. doi: 10.1080/10522158.2014.888696

[40] Ajzen I. The theory of planned behavior. Organ Behav Hum Decis Process. 1991;50: 179–211. doi: 10.1016/0749-5978(91)90020-T

[41] Dishion TJ, Mun CJ, Drake EC, Tein JY, Shaw DS, Wilson M. A transactional approach to preventing early childhood neglect: The Family Check-Up as a public health strategy. Dev Psychopathol. 2015;27: 1647–1660. doi: 10.1017/S0954579415001005 26535950 PMC4801330

[42] Alvarez M de la C, García BH, Navarro Flores CM, Vázquez AL, Lara J, Domenech Rodríguez MM. Parent Training Interventions. Reference Module in Biomedical Sciences. Elsevier; 2021. doi: 10.1016/B978-0-12-818872-9.00030–3

[43] Vázquez AL, Villodas MT. Racial/ethnic differences in caregivers’ perceptions of the need for and utilization of adolescent psychological counseling and support services. Cult Divers Ethn Minor Psychol. 2018;25: 323–330. doi: 10.1037/cdp0000255 30570291

[44] Vázquez AL, Navarro Flores CM, Alvarez M de la C, Domenech Rodríguez MM. Latinx caregivers’ perceived need for and utilization of youth telepsychology services during the coronavirus pandemic. J Latinx Psychol. 2021. doi: 10.1037/lat0000192

