# Supplementary figures and images for: High value correlates of caregiver reported counseling service need and utilization for adolescents at-risk for childhood maltreatment and neglect
Source: PLoS One. 2021 Oct 1;16(10):e0258082. doi: 10.1371/journal.pone.0258082 (PMC8486129; doi:10.1371/journal.pone.0258082)

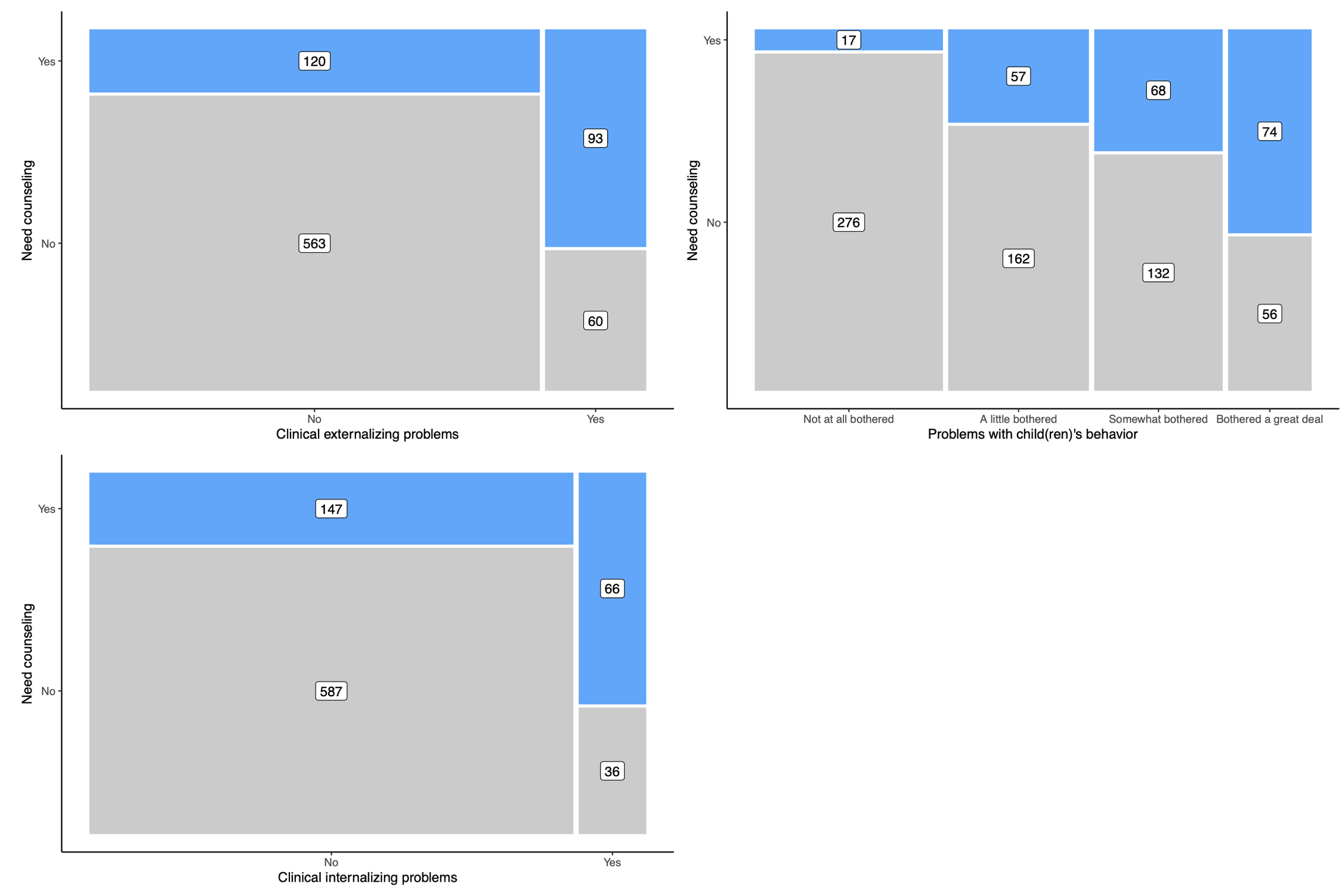

Supplement: S1 Fig — (TIF) [file pone.0258082.s001.tif]

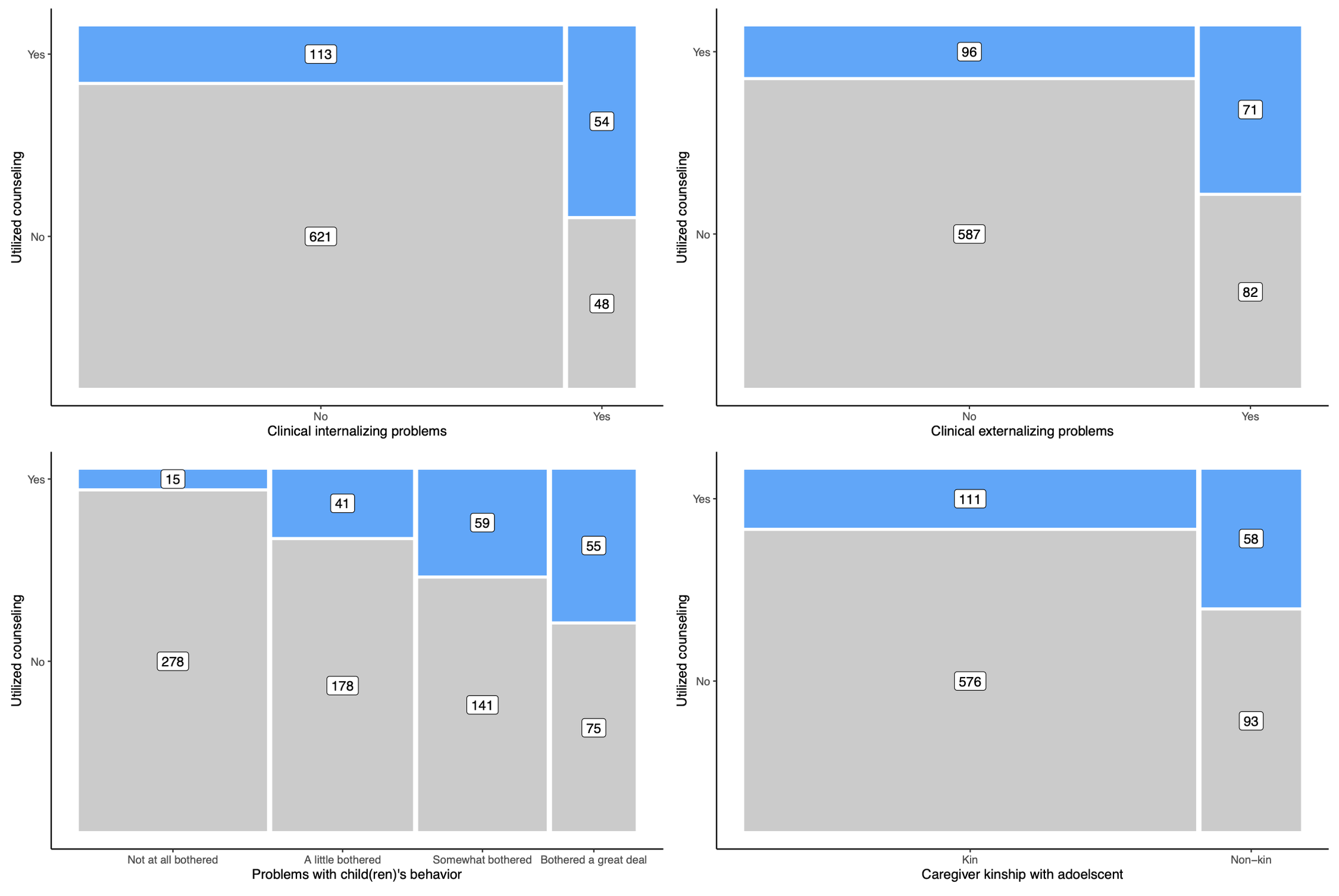

Supplement: S2 Fig — (TIF) [file pone.0258082.s002.tif]
